# Deep vein thrombosis and pulmonary embolism: a prospective, observational study to evaluate diagnostic performance of the Tina-quant D-Dimer Gen.2 assay

**DOI:** 10.3389/fcvm.2023.1142465

**Published:** 2023-12-18

**Authors:** Thomas Bertsch, Wilhelm Behringer, Sabine Blaschke, Richard Body, Simon Davidson, Mirco Müller-Olling, Ge Guo, Anna Rieger, Annika Wahl, Daniel Horner, Yuli Sun, Lucia Turnes, Ulrich Sonner, Michael Hoffmann

**Affiliations:** ^1^ Institute of Clinical Chemistry, Laboratory Medicine and Transfusion Medicine, Nuremberg General Hospital, Paracelsus Medical University, Nuremberg, Germany; ^2^ Department of Emergency Medicine, Faculty of Medicine, University of Jena, Jena, Germany; ^3^ Emergency Department, University Medical Center Göttingen, Göttingen, Germany; ^4^ Division of Cardiovascular Sciences, Core Technology Facility, University of Manchester, Manchester, United Kingdom; ^5^ Emergency Department, Manchester University NHS Foundation Trust, Manchester, United Kingdom; ^6^ Roche Diagnostics International Ltd, Rotkreuz, Switzerland; ^7^ Roche Diagnostics GmbH, Mannheim, Germany; ^8^ Roche Diagnostics, Indianapolis, IN, United States; ^9^ Roche Diagnostics GmbH, Penzberg, Germany; ^10^ Emergency Department, Salford Royal NHS Foundation Trust, Salford, United Kingdom; ^11^ Division of Infection, Immunity and Respiratory Medicine, University of Manchester, Manchester, United Kingdom; ^12^ Agent Representing Roche Diagnostics GmbH, Penzberg, Germany

**Keywords:** coagulation assay, cobas t 711, D-Dimer, deep vein thrombosis, pulmonary embolism

## Abstract

**Background:**

D-Dimer testing is a diagnostic tool for exclusion of deep vein thrombosis (DVT) and pulmonary embolism (PE). This study evaluated the diagnostic performance of the Tina-quant® D-Dimer Gen.2 assay (Roche Diagnostics International Ltd, Rotkreuz, Switzerland) in patients with low/intermediate pre-test probability of DVT/PE using standard, age-, and clinical probability-adjusted cut-offs.

**Methods:**

In this prospective, observational, multicenter study (July 2017–August 2019), plasma samples were collected from hospital emergency departments and specialist referral centers. DVT/PE was diagnosed under hospital standard procedures and imaging protocols. A standard D-dimer cut-off of 0.5 µg fibrinogen equivalent units (FEU)/ml was combined with the three-level Wells score; cut-offs adjusted for age (age × 0.01 µg FEU/ml for patients >50 years) and clinical probability (1 µg FEU/ml for low probability) were also evaluated. An assay comparison was conducted in a subset of samples using the Tina-quant D-Dimer Gen.2 assay and the previously established routine laboratory assay, STA-Liatest D-Di Plus assay (Stago Deutschland GmbH, Düsseldorf, Germany).

**Results:**

2,897 patients were enrolled; 2,516 completed the study (DVT cohort: 1,741 PE cohort: 775). Clinical assessment plus D-dimer testing using the standard cut-off resulted in 317 (DVT) and 230 (PE) false positives, and zero (DVT) and one (PE) false negatives. Negative predictive value (NPV) was 100.0% (95% confidence interval [CI]: 99.7%–100.0%) and 99.8% (95% CI: 98.8%–100.0%) for DVT and PE, respectively. After age-adjustment, NPV was 99.9% (95% CI: 99.6%–100.0%) and 99.1% (95% CI: 97.8–99.7) for DVT and PE, respectively. False positive rates decreased (>50%) in clinical probability-adjusted analyses vs. primary analysis. In the assay comparison, the performances of the two assays were comparable.

**Conclusion:**

The Tina-quant D-Dimer Gen.2 assay and standard D-dimer cut-off level combined with the three-level Wells score accurately identified patients with a very low probability of DVT/PE.

## Introduction

Deep vein thrombosis (DVT) and pulmonary embolism (PE), clinical manifestations of venous thromboembolism (VTE), cause significant morbidity and mortality worldwide (1–3). In the United States, VTE is diagnosed in approximately 0.2% of the population and though, in recent years, the mortality rate of patients with VTE has declined, it can still be up to 26% (2). Multiple factors contribute to venous thrombotic risk, such as abnormalities in blood coagulation factors, cancer, and age, the latter being the strongest risk factor (4).

D-dimer and fibrin X-oligomers are biomarkers for the simultaneous activation of coagulation and fibrinolysis that occurs in the presence of thrombosis (5). High levels of D-dimer and X-oligomer fibrin degradation products are indicative of thrombotic risk for DVT and PE; conversely, normal levels identify individuals with a low probability of DVT or PE. International guidelines recommend D-dimer testing in conjunction with clinical presentation, pre-test probability assessment, and imaging, as an important diagnostic tool for DVT and PE (6, 7). Clinical probability assessment, such as that provided by the two- or three-level Wells score, acts as a clinical decision aid for patients suspected of having DVT or PE (8–10). The three-level Wells score is based on clinical symptoms and known risk factors for VTE and categorizes patients as being at either low, intermediate, or high risk of DVT (<1, 1–2, or ≥3 points, respectively) or PE (0–1, 2–6, or >6 points, respectively). When used alongside a highly sensitive D-dimer assay, patients at low or intermediate risk can undergo D-dimer testing in the emergency department to determine the likelihood that a clot is present, rather than undergo definitive investigation. A finding of a normal D-dimer level (<0.5 µg fibrinogen equivalent units [FEU]/ml, with consideration of the use of age-specific cut-offs to increase specificity) (11) can identify patients with a very low probability of DVT or PE and circumvents the need for costly investigative procedures (6, 12, 13). An elevated D-dimer level is not itself considered to be diagnostic of DVT or PE, but rather indicates that imaging is required (13, 14).

Commercially available D-dimer assays generally fall into one of three main categories: qualitative whole blood agglutination assays, which have low sensitivity but high specificity; quantitative enzyme-linked immunosorbent (ELISA) or immunofluorescent assays, and latex agglutination assays, which have high sensitivity but low specificity (15); and latex-enhanced immunoturbidimetric assays, which have the advantages of comparable high sensitivity to ELISA assays, intermediate specificity, the capability to be fully automated, and the most rapid quantification of plasma D-dimer levels for patients presenting to the emergency department (16–18).

Clinicians need to be aware of the considerable degree of variation in the performance characteristics across different D-dimer assays to be able to interpret test results. This is particularly important if the assay cut-off is moderated according to clinical pre-test probability or patient age (17, 19), and assays should be tested and validated in clinical trials or prospective management studies (16). The use of age-adjustment is relevant as D-dimer levels are naturally increased in patients aged >50 years (20). Highly sensitive D-dimer assays benefit from the use of the three-level Wells score over the two-level score, with subsequently fewer patients in need of further imaging, thereby reducing both costs and unnecessary exposure of patients to ionizing radiation. The use of age- and clinical probability-adjustments has been shown to have superior specificity and clinical utility compared with the standalone D-dimer interpretation (21).

The Tina-quant® D-Dimer Gen.2 assay (Roche Diagnostics International Ltd, Rotkreuz, Switzerland)^1^ is a latex-enhanced immunoturbidimetric assay for the quantitative immunologic determination of D-dimer and X-oligomers in citrated plasma samples. Prospective management studies support the use of the Tina-quant D-Dimer assay to identify patients with a very low probability of DVT and PE^2^. The aim of this study was to evaluate the diagnostic performance of the Tina-quant D-Dimer Gen.2 assay for identifying patients with a very low probability of proximal DVT or PE in a large cohort of patients with a low or intermediate pre-test clinical probability assessment, using the standard cut-off of 0.5 µg FEU/ml, an age adjusted cut-off, and a clinical probability-adjusted cut-off.

## Materials and methods

### Study design

This was a prospective, observational, multicenter study conducted between July 3, 2017 and August 28, 2019. All plasma samples were collected from European hospital emergency departments and specialist referral centers (Supplementary Table S1). All sites were experienced in the clinical diagnosis and treatment of DVT and/or PE and were familiar with the three-level Wells score (8–10); Supplementary Table S2) as a clinical decision rule for assessment. Ethical approval was obtained from institutional review boards for all study sites. Written informed consent was obtained from all eligible patients. Protection of privacy regulations were followed as per European Union General Data Protection Regulation (GDPR) 2016/679. The study was performed in accordance with Directives 90/835/EEC and 93/42/EEC.

### Study objectives and endpoints

The primary aim of this study was to evaluate the diagnostic performance of the Tina-quant D-Dimer Gen.2 assay for identifying patients with a very low probability of DVT or PE presenting to the emergency department with suspected DVT and/or PE, using a standard cut-off of 0.5 µg FEU/ml D-dimer in conjunction with a low or intermediate pre-test probability according to the relevant three-level Wells criteria. An exploratory objective was to evaluate the diagnostic performance of the assay using an age-adjusted cut-off for patients aged >50 years. A *post hoc* exploratory objective to evaluate the diagnostic performance using a clinical probability-adjusted cut-off of 1 µg FEU/ml for patients with low risk was also included.

The pre-defined primary endpoint was DVT and/or PE confirmed by imaging, or DVT- and/or PE-related death at 90 days. For the DVT cohort, imaging-confirmed proximal DVT (at or above the level of the trifurcation area) was used as the primary endpoint for analysis; for the PE cohort, imaging-confirmed PE was used as the primary endpoint for analysis.

The secondary aim of this study was to compare the basic performance characteristics (negative predictive value [NPV], positive predictive value [PPV], sensitivity, and specificity) of the Tina-quant D-Dimer Gen.2 assay with a D-dimer assay that was established for routine measurements at one of the study sites.

### Patients

Eligible patients were aged ≥18 years, hemodynamically stable, had a Wells score indicating a low or intermediate pre-test probability for DVT (≤2) or PE (≤6) based on the respective three-level scoring, and had at least one lead symptom for DVT or PE (or other documented reason for enrolment). Participant enrollment was consecutive.

Exclusion criteria were: symptoms of DVT or PE for >7 days; a high pre-test probability for DVT or PE according to three-level Wells score (Supplementary Table S2); previous DVT and/or PE; use of unfractionated heparin, low-molecular-weight heparin, or oral anticoagulants in the past 4 days (for vitamin K antagonists, within the past 4 weeks); contraindications for any required diagnostic imaging; use of thrombolytic agents within the past 7 days; patients already hospitalized (for any reason); a concomitant clinical suspicion of PE (for patients with DVT only—in such cases, patients were enrolled in the PE cohort); self-reported pregnancy; impossibility for follow-up by phone or email; or life expectancy of <3 months.

DVT and/or PE were diagnosed in symptomatic patients by physicians according to reference standard procedures and imaging protocols, in line with national guidance^3^ and established evidence (22). Patients were categorized as having low and intermediate pre-test probability by means of Wells scores of <1 (DVT) and <2 (PE), and 1–2 (DVT) and 2–6 (PE), respectively. All patients were followed-up for 90–111 days after discharge from the emergency department to verify the clinical diagnosis and record any adverse events. For the purpose of analysis, patients were assigned to DVT or PE (with/without concomitant DVT) cohorts.

### Sample processing

Each study site prospectively collected patient blood samples by preparing citrated plasma from a single blood draw for each enrolled patient. Plasma samples were frozen and shipped on dry ice to a central laboratory for D-dimer testing. Samples were pseudonymized and stored at −70°C until testing. For D-dimer measurements, all samples were randomized. Analyses were performed using a cobas® t 711 analyzer (Roche Diagnostics International Ltd, Rotkreuz, Switzerland) at a central laboratory used as the single measurement site (Nuremberg, Germany). A subset of samples (DVT: *N* = 951; PE: *N* = 555 for PE), for which sufficient volume was left after the measurements for determination of clinical performance, was used in an exploratory comparison with the STA-Liatest D-Di Plus assay (Stago Deutschland GmbH, Düsseldorf, Germany), which was already established for routine measurements in the Nuremberg laboratory. For the assay comparison, samples were first measured with the Tina-quant D-Dimer Gen.2 assay using the cobas t 711 analyzer, followed within 1 h by a second measurement with the STA-Liatest D-Di Plus assay on the STA-R MAX analyzer (Stago Deutschland GmbH, Düsseldorf, Germany), according to manufacturer's instructions. Tina-quant D-Dimer Gen.2 assay.

### Tina-quant D-Dimer Gen.2 assay

The Tina-quant D-Dimer Gen.2 assay is based on latex particles coated with monoclonal antibodies (F[ab']2 fragments) to the D-dimer epitope. Addition of a human citrated plasma sample containing D-dimer results in the formation of antigen/antibody complexes that lead to an increase in turbidity; the aggregate is measured using turbidimetry. Change in absorbance over time is dependent on the concentration of D-dimer in the sample. In this study, the D-dimer level of patients was measured *post hoc* in batches and results paired to clinical diagnosis. Assay data were directly captured using WinCAEv (Mannheim, Germany), a 21 Code of Federal Regulations Part 11 compliant electronic data capture software.

### Data analysis

A sample size calculation was performed. With a projected lower prevalence of DVT/PE of 8%, it was planned to collect 70 total positive cases in each cohort to meet sensitivity criteria of >97% with a maximum of two false negatives. The observed prevalence of DVT/PE was 3%, lower than the expected 8%, so the number of maximally allowed false negatives in the DVT cohort was reduced to zero. Data were analyzed according to a statistical analysis plan and using SAS software (version 9.4) and R software (versions 3.4.0 and 3.5.1). Diagnostic performance of the Tina-quant D-Dimer Gen.2 and STA-Liatest D-Di Plus assays was assessed by calculating NPV, PPV, sensitivity and specificity, with 95% exact Clopper-Pearson confidence intervals (CIs). This was performed for the DVT and PE cohorts separately, and in combination as the overall VTE population.

Based on the calculated sensitivity and specificity of the assay, the positive likelihood ratios (LR+) and negative likelihood ratios (LR–) were calculated, and CIs for the likelihood ratios were calculated using the Log method (23).

Diagnostic performance was also calculated using age- and clinical probability-adjusted cut-offs, in accordance with published methodology (20). The age-adjusted cut-off was applied for patients aged >50 years and determined by multiplying patient age by 0.01 µg FEU/ml (e.g., aged 55 years × 0.01 = cut-off of 0.55 µg FEU/ml). The effect of age-adjustment on the whole population was analyzed, as was the effect within each decade for patients aged >50 years. For the clinical probability-adjusted analysis, a higher cut-off of 1 µg FEU/ml was applied to patients assessed as being at low risk of DVT and/or PE according to the three-level Wells score.

All analyses were carried out by the Biostatistics group within Roche Diagnostics (Indianapolis, IN, USA, and Penzberg, Germany).

## Results

### Patients

A total of 2,897 patients were enrolled; 1,982 patients (68.4%) were enrolled to the DVT cohort and 915 (31.6%) to the PE cohort (Supplementary Figure S1). A total of 2,516 eligible patients completed the study (≥90 days' follow-up): 87.8% (1,741/1,982) of patients in the DVT cohort and 84.7% (775/915) of patients in the PE cohort. Between 90 and 111 days post-enrollment, 6.2% (108/1,741) of patients in the DVT cohort (3.4% [59/1,741] had proximal DVT) and 10.2% (79/775) of patients in the PE cohort received a positive diagnosis following imaging-confirmed DVT and/or PE, or DVT- and/or PE-related death. Baseline characteristics of the patients are shown in Table 1. In total, 57.2% (996/1,741) of patients in the DVT cohort and 69.9% (542/775) of patients in the PE cohort were aged >50 years; the majority of patients eventually diagnosed with DVT/PE were aged >50 years (79.7% DVT, 82.3% PE).

**Table 1:** Patient demographics and baseline characteristics.

	DVT cohort			PE cohort		
	Suspected	Diagnosed	Total	Suspected	Diagnosed	Total
*N*	1,682	59	1,741	696	79	775
Age						
Mean (SD)	53 (14.1)	62 (12.6)	53 (14.1)	58 (16.6)	66 (14.0)	58 (16.6)
Median (range)	53 (18–92)	64 (33–88)	54 (18–92)	59 (18–91)	69 (25–87)	61 (18–91)
*P* 0.05–*P* 0.95	29–76	37–80	29–76	27–82	35–83	28–82
Age group, years, *N* (%)						
≤50	733 (43.6)	12 (20.3)	745 (42.8)	219 (31.5)	14 (17.7)	233 (30.1)
>50	949 (56.4)	47 (79.7)	996 (57.2)	477 (68.5)	65 (82.3)	542 (69.9)
Sex, *N* (%)						
Female	1,140 (67.8)	17 (28.8)	1,157 (66.5)	394 (56.6)	44 (55.7)	438 (56.5)
Male	542 (32.2)	42 (71.2)	584 (33.5)	302 (43.4)	35 (44.3)	337 (43.5)
Race, *N* (%)						
Caucasian	1,635 (97.2)	55 (93.2)	1,690 (97.1)	655 (94.1)	78 (98.7)	733 (94.6)
Other	10 (0.6)	2 (3.4)	12 (0.7)	3 (0.4)	0 (0.0)	3 (0.4)
Not reported	37 (2.2)	2 (3.4)	39 (2.2)	38 (5.5)	1 (1.3)	39 (5.0)
Malignancy (via Wells score), *N* (%)						
No	1,676 (99.6)	59 (100.0)	1,735 (99.7)	676 (97.1)	76 (96.2)	752 (97.0)
Yes	6 (0.4)	0 (0.0)	6 (0.3)	20 (2.9)	3 (3.8)	23 (3.0)

DVT, deep vein thrombosis; PE, pulmonary embolism; *P* x, x percentile; SD, standard deviation.

### Tina-quant D-Dimer Gen.2 assay: clinical performance

In the overall patient population, NPV and sensitivity for identifying patients with a very low probability of DVT and/or PE were high: 100.0% (95% CI: 99.7%–100.0%) and 99.3% (95% CI: 96.0%–100.0%), respectively (Supplementary Table S3). Overall PPV and specificity were 20.0% (95% CI: 17.1%–23.2%) and 77.0% (75.3%–78.7%), respectively. Similar results were observed in patients with low pre-test probability and in those with intermediate pre-test probability (Supplementary Table S3).

Analyses stratified by cohort and by pre-test probability classification showed diagnostic performance results consistent with the overall findings (Table 2). The prevalence of DVT and PE in patients with low and intermediate pre-test probability was 0% (DVT) and 2.2% (PE), and 3.7% (DVT) and 13.6% (PE), respectively. Clinical assessment combined with D-dimer testing resulted in 317 false positives and no false negatives in the DVT cohort (*N *= 1,741), and 230 false positives and one false negative in the PE cohort (*N* = 775). NPV was 100.0% (95% CI: 99.7%–100.0%) and 99.8% (95% CI: 98.8%–100.0%) for DVT and PE, respectively. Sensitivity was 100.0% (95% CI: 93.9%–100.0%) and 98.7% (95% CI: 93.2%–100.0%) for DVT and PE, respectively. PPV ranged from 0.0% (95% CI: 0.0%–17.7%) to 28.5% (95% CI: 23.1%–34.5%) and specificity from 61.2% (95% CI: 56.6–65.6) to 86.4% (95% CI: 79.6%–91.6%).

**Table 2:** Diagnostic performance of the Tina-quant D-Dimer Gen.2 assay by cohort and pre-test probability classification (*N* = 2,516).

	True positive, *N*	False positive, *N*	False negative, *N*	True negative, *N*	NPV, % (95% CI)	PPV, % (95% CI)	Sensitivity, % (95% CI)	Specificity, % (95% CI)	LR + (95% CI)	LR − (95% CI)
DVT cohort										
All (*N* = 1,741)	59	317	0	1,365	100.0 (99.7–100.0)	15.7 (12.2–19.8)	100.0 (93.9–100.0)	81.2 (79.2–83.0)	5.3 (4.8–5.9)	0.0 (N/A)
Low pre-test probability (*N* = 140)	0	19	0	121	100.0 (97.0–100.0)	0.0 (0.0–17.7)	N/A (N/A)	86.4 (79.6–91.6)	0.0 (N/A)	N/A (N/A)
Intermediate pre-test probability (*N* = 1,601)	59	298	0	1,244	100.0 (99.7–100.0)	16.5 (12.8–20.8)	100.0 (93.9–100.0)	80.7 (78.6–82.6)	5.2 (4.7–5.7)	0.0 (N/A)
PE cohort										
All (*N* = 775)	78	230	1^a^	466	99.8 (98.8–100.0)	25.3 (20.6–30.6)	98.7 (93.2–100.0)	67.0 (63.3–70.4)	3.0 (2.7–3.3)	0.02 (0.0–0.1)
Low pre-test probability (*N* = 230)	5	47	0	178	100.0 (98.0–100.0)	9.6 (3.2–21.0)	100.0 (47.8–100.0)	79.1 (73.2–84.2)	4.8 (3.7–6.2)	0.0 (N/A)
Intermediate pre-test probability (*N* = 545)	73	183	1^a^	288	99.7 (98.1–100.0)	28.5 (23.1–34.5)	98.7 (92.7–100.0)	61.2 (56.6–65.6)	2.5 (2.3–2.9)	0.0 (0.0–0.2)

CI, confidence interval; DVT, deep vein thrombosis; ECG, electrocardiogram; LR+, positive likelihood ratio; LR−, negative likelihood ratio; N/A, not applicable; NPV, negative predictive value; PE, pulmonary embolism; PPV, positive predictive value.

^a^ Female, 82 years, Caucasian; 170 cm, 69 kg; enrolled on December 5, 2018; presented directly to the emergency department (not by referral). No DVT lead symptoms; PE lead symptoms: tachycardia, hypotension. Vital signs: blood pressure 90/60, heart rate 120 bpm, respiratory rate 19 breaths per minute; oxygen saturation 87%; no intranasal oxygen supplementation performed; Wells score PE 4.5 (based on tachycardia and no alternative diagnosis better explaining the illness; no malignancies under treatment, treated within last 6 months or palliative therapy). Conventional 12-lead ECG; sinus tachycardia; electrical heart axis directed right; bundle branch block normal; no left-axis deviation; S1S2S3 and S1Q3R3 types: both not reported; overall ECG: non-specific (accepted deviation from the norm, with lowest likelihood of ischemia or PE).

The LR + for DVT and PE were 5.3 (95% CI: 4.8–5.9) and 3.0 (95% CI: 2.7–3.3), respectively (Table 2). The LR– for DVT and PE were 0.0 (95% CI: not available) and 0.02 (95% CI: 0.0–0.1), respectively.

### Age-adjusted analysis

In total, 1,538 patients were aged >50 years (996 DVT cohort; 542 PE cohort). An exploratory analysis using an age-adjusted D-dimer cut-off, determined by multiplying patient age by 0.01 µg FEU/ml, was performed in these patients and assay performance parameters were calculated.

Specificity was higher in this age-adjusted analysis (Table 3) compared with the primary analysis, with false positive rates of approximately 13% and 22%, respectively. However, the sensitivity was lower in the PE group (93.7% [95% CI: 85.8–97.9]) and false negatives increased from 0% to 0.06% (1/1,741) in the DVT cohort and from 0.1% (1/775) to 0.6% (5/775) in the PE cohort. When patients aged >50 years were grouped by decade of life, false positive rates were lower in the age-adjusted analysis vs. the primary analysis in every age-group category and appeared to decrease with increasing patient age (Supplementary Table S4).

**Table 3:** Diagnostic performance of the Tina-quant D-Dimer Gen.2 assay in the exploratory age-adjusted analysis^a^ (*N* = 2,516).

Group	True positive, *N*	False positive, *N*	False negative, *N*	True negative, *N*	NPV, % (95% CI)	PPV, % (95% CI)	Sensitivity, % (95% CI)	Specificity, % (95% CI)	LR + (95% CI)	LR − (95% CI)
DVT cohort (*N* = 1,741)	58	232	1	1,450	99.9 (99.6–100.0)	20.0 (15.6–25.1)	98.3 (90.9–100.0)	86.2 (84.5–87.8)	7.1 (6.3–8.1)	0.0 (0.0–0.1)
PE cohort (*N* = 775)	74	170	5	526	99.1 (97.8–99.7)	30.3 (24.6–36.5)	93.7 (85.8–97.9)	75.6 (72.2–78.7)	3.8 (3.3–4.4)	0.1 (0.0–0.2)

CI, confidence interval; DVT, deep vein thrombosis; FEU, fibrinogen equivalent units; LR+, positive likelihood ratio; LR−, negative likelihood ratio; NPV, negative predictive value; PE, pulmonary embolism; PPV, positive predictive value.

^a^ A cut-off of 0.5 µg FEU/ml was used for patients aged ≤50 years; an age-adjusted cut-off in patients aged >50 years was determined by multiplying age by 0.01 µg FEU/ml (e.g., aged 55 years × 0.01 = age-adjusted cut-off of 0.55 µg FEU/ml).

Using age-adjustment, the number of false negatives increased from zero to one and one to five for the DVT and PE cohorts, respectively. The false negative patient for DVT was a 59-year old male with a D-dimer level of 0.552 µl/ml. The patient's test result became negative when the cut-off was adjusted to 0.59 µl/ml; he was diagnosed with proximal and distal DVT. The additional false negative patients for PE were: a 81-year old male with a D-Dimer level of 0.697 µl/ml; a 73-year old male with a D-dimer level of 0.664 µl/ml; a 79-year old female with a D-dimer level of 0.727 µl/ml; and a 72-year old female with a D-dimer level of 0.705 µl/ml.

### Clinical probability-adjusted analysis

A *post hoc* exploratory analysis using a cut-off of 1 µg FEU/ml for the low pre-test probability group is presented in Table 4. This approach resulted in a greater than 50% decrease in false positives and an increase in assay specificity in both the DVT and PE cohorts compared with the primary analysis, without any missed cases of DVT or PE.

**Table 4:** Diagnostic performance of the Tina-quant D-Dimer Gen.2 assay in the *post hoc* exploratory clinical probability-adjusted analysis^a^ (*N* = 370).

Group	True positive, *N*	False positive, *N*	False negative, *N*	True negative, *N*	NPV, % (95% CI)	PPV, % (95% CI)	Sensitivity, % (95% CI)	Specificity, % (95% CI)	LR + (95% CI)	LR − (95% CI)
DVT cohort (*N* = 140)^b^	0	5	0	135	100.0 (97.3–100.0)	0.0 (0.0–52.2)	N/A (N/A)	96.4 (91.9–98.8)	N/A (N/A)	N/A (N/A)
PE cohort (*N* = 230)^b^	5	21	0	204	100.0 (98.2–100.0)	19.2 (6.6–39.4)	100.0 (47.8–100.0)	90.7 (86.1–94.1)	10.7 (7.1–16.1)	0.0 (N/A)

CI, confidence interval; DVT, deep vein thrombosis; FEU, fibrinogen equivalent units; LR+, positive likelihood ratio; LR−, negative likelihood ratio; N/A, not applicable; NPV, negative predictive value; PE, pulmonary embolism; PPV, positive predictive value.

^a^ A cut-off of 1 µg FEU/ml was applied for patients designated to have a low clinical probability of DVT and/or PE according to the Wells three-level test.

^b^ Wells score: Low.

The sensitivity and specificity of the Tina-quant D-Dimer Gen.2 assay based on a cut-off of 1 µg/ml in the DVT cohort were 66.67% (95% CI: 34.89–90.08) and 97.00% (95% CI: 95.49–98.11) in patients aged ≤50 years, and 82.98% (95% CI: 69.19–92.35) and 90.09% (95% CI: 88.02–91.92) in patients aged >50 years. The sensitivity and specificity of the Tina-quant D-Dimer Gen.2 assay based on a cut-off of 1 µg/ml in the PE cohort were 92.86% (95% CI: 66.13–99.82) and 94.52 (90.62–97.14) in patients aged ≤50 years, and 84.62 (73.52–92.37) and 77.99 (74.00–81.63) in patients aged >50 years.

### Safety

No serious adverse events, adverse device effects, or serious adverse device effects were reported. Two minor adverse events occurred in 2 patients during venipuncture: one small hematoma and one burst vein.

### Assay comparison

The Tina-quant D-Dimer Gen.2 assay was compared with the established routine laboratory assay, STA-Liatest D-Di Plus, using a subset of plasma samples. As shown in Table 5, the performances of the two assays were comparable. Sensitivity with the Tina-quant D-Dimer Gen.2 assay measured on the cobas t 711 analyzer was 95.3% (95% CI: 89.3%–98.5%) for DVT and 100.0% (95% CI: 95.2%–100.0%) for PE; for the STA-Liatest D-Di Plus assay, respective values were 93.1% (95% CI: 86.4%–97.2%) and 98.6% (95% CI: 92.6%–100.0%). Specificity with the Tina-quant D-Dimer Gen.2 assay on the cobas t 711 analyzer was 69.5% (95% CI: 66.3%–72.5%) for DVT and 53.4% (95% CI: 48.9%–57.9%) for PE; for the STA-Liatest D-Di Plus assay, respective values were 76.5% (95% CI: 74.2%–78.6%) and 60.7% (95% CI: 57.0%–64.4%).

**Table 5:** Assay comparison of the Tina-quant D-Dimer Gen.2 assay with the STA-Liatest D-Di plus assay (established for routine measurements in the laboratory) using a subset of plasma samples.

Group	Instrument	Samples (*N*)	NPV, % (95% CI)	PPV, % (95% CI)	Sensitivity, % (95% CI)	Specificity, % (95% CI)
DVT cohort	cobas t 711	1,003	99.2 (98.2–99.7)	26.9 (22.5–31.7)	95.3 (89.3–98.5)	69.5 (66.3–72.5)
	STA-R Max	1,623	99.4 (98.8–99.8)	21.0 (17.3–25.0)	93.1 (86.4–97.2)	76.5 (74.2–78.6)
PE cohort	cobas t 711	569	100.0 (98.6–100.0)	24.6 (19.9–29.8)	100.0 (95.2–100.0)	53.4 (48.9–57.9)
	STA-R Max	758	99.8 (98.7–100.0)	21.1 (16.9–25.8)	98.6 (92.6–100.0)	60.7 (57.0–64.4)

CI, confidence interval; DVT, deep vein thrombosis; NPV, negative predictive value; PE, pulmonary embolism; PPV, positive predictive value.

There were 200 discordant samples, all from patients diagnosed as VTE-negative. Of the 200 samples, 134 and 66 samples were from the DVT and PE cohorts, respectively.

## Discussion

Our findings show that the Tina-quant D-Dimer Gen.2 assay has high NPV and high sensitivity (100.0% [95% CI: 99.7%–100.0%] and 99.3% [95% CI: 96.0%–100.0%], respectively) for identifying patients with a very low probability of DVT and/or PE in conjunction with the three-level Wells score. If used in accordance with international standards [see text footnote 3, (22)] for investigation of DVT and PE, this assay could have prevented the need for further imaging in approximately 299 (80.0%) patients with a low-risk score, and 1,532 (71.4%) patients with an intermediate score in this study, with only a single false negative result (see the column of true negatives in Table 2). Testing with the Tina-quant D-Dimer Gen.2 assay is a reliable method for identifying patients with a very low probability of DVT and/or PE. LR– values were 0 or very close to 0 in both cohorts and pre-test probability groups, strongly indicating that patients with a negative result using the Tina-quant D-Dimer Gen.2 assay have a very low probability of DVT or PE. In the PE cohort, it should also be noted that the sample size was smaller and the LR– had wide CIs. When compared with the STA-Liatest D-Di Plus assay using a subset of plasma samples, the Tina-quant D-Dimer Gen.2 assay demonstrated comparable performance. The sensitivity of the STA-Liatest D-Di Plus assay was slightly lower for DVT and slightly higher for PE compared with the manufacturer's package insert (DVT: *N* = 980, 100.0% [95% CI: 95.8%–100.0%]; PE: *N* = 1,060, 97.6% [95% CI: 91.7%–99.7%]); data were based on clinical studies similar to this study, which enrolled patients with non-high pre-test probability (24, 25).

The strengths of the study include its prospective design and the large sample size; however, patients at high risk of DVT and PE were excluded, as per predefined criteria. These patients were excluded as the primary aim was to rule out these diagnoses and it is widely accepted in clinical practice for patients who are at high risk according to the Wells score to proceed directly to imaging, regardless of the D-dimer concentration (8–10). In this study, the prevalence of PE was 2.2% and 13.6% in patients with low and intermediate pre-test probability, respectively. These values are comparable with those reported by Ceriani et al*.* (26) in a meta-analysis of 14 studies using the three-level Wells test, who found that the pooled prevalence for out-patients with low and intermediate pre-test probability was 2.9% and 15.8%, respectively. Moreover, an assay comparison using an established laboratory measurement system demonstrated comparable analytical results when using the Tina-quant D-Dimer Gen.2 assay measured on the cobas t 711 analyzer.

The YEARS study (27) also showed that a simplified diagnostic management of suspected PE by combining the YEARS clinical decision rule (clinical signs of DVT, hemoptysis, and whether PE is the most likely diagnosis) with fixed D-dimer thresholds was able to identify patients with a very low probability of PE and decrease the need for further imaging in 48% vs. 34% of patients when compared with the conventional two-level Wells rule. A secondary analysis of the YEARS study (28) also showed that the YEARS algorithm, which focused on the YEARS criteria and D-dimer cut-offs (500 or 1,000 ng/ml), resulted in a faster diagnosis of PE compared with the conventional algorithm, which managed patients based on the Wells clinical decision rule as well as age-adjusted D-dimer cut-offs (500 ng/ml for patients aged <50 years; patients' age × 10 ng/ml for patients aged ≥50 years).

Notably, exploratory analyses using age-adjusted ranges for predicting the presence of DVT/PE in patients with low or intermediate pre-test probability reduced false positive rates by approximately 25%. Therefore, assuming no further imaging with this strategy, it may be possible to obviate the need for further imaging in 1,976 (78.5%) patients. This strategy increased the number of false negatives for the PE cohort from 0 to 1 in our study. However, the utility of age-adjusted D-dimer thresholds for PE has been highlighted in prospective studies including the ADJUST-PE study (29) and the RELAX-PE study (30). The diagnostic performance of the assay should be further considered in the context of its health economic potential; one study by Blondon et al. concluded that use of an age-adjusted D-dimer cut-off had the potential of cost savings >$80 million per year for the United States health system (31). In support, a recent retrospective study also emphasized the cost-effectiveness of diagnostic strategies using age-adjusted cut-off levels to rule out VTE (32). Encouragingly, our *post hoc* exploratory analysis using a cut-off of 1 μg FEU/ml in patients with a low pre-test probability further reduced false positive rates by >50% without any increase in false negative results. These reductions may potentially lead to fewer invasive procedures and additional tests for patients, resulting in both time and cost savings, as well as less inconvenience for patients.

Age-adjustment for D-dimer is increasingly being utilized for the prediction of DVT/PE (33), with clinical guidelines gradually adopting this approach. However, evidence in favor of age-adjusted analysis is incomplete; new assays need to be better defined and more evidence is needed to validate the cut-offs. An extensive literature review by the National Institute for Health and Care Excellence (see text footnote 3) found that age-adjusted D-dimer cut-off points resulted in a large reduction of false positives, which could decrease imaging rates; however, they also found a small increase in false negatives, therefore potentially missing disease cases. Further work is required to determine whether an assay with higher precision might identify a greater proportion of patients with a very low probability of DVT or PE by adopting a lower cut-off.

## Conclusions

The Tina-quant D-Dimer Gen.2 assay in combination with a low/intermediate pre-test probability according to three-level Wells criteria identified patients with a very low probability of DVT and/or PE (NPV: 100.0% [95% CI: 99.7%–100.0%] for DVT and 99.8% [95% CI: 98.8%–100.0%] for PE) with high sensitivity (100.0% for DVT and 98.7% for PE). The assay also performed well with age-adjusted and clinical probability-adjusted cut-offs.

## Data Availability

The raw data supporting the conclusions of this article will be made available by the authors, without undue reservation, dependent on legal agreements between the sponsor, study sites, and participants.

## Footnotes

^1^ https://pim-eservices.roche.com/eLD/api/downloads/f0ed7cae-7227-ed11-1691-005056a772fd?countryIsoCode=gb

^2^ https://pim-eservices.roche.com/eLD/api/downloads/d8174e0e-b48f-ec11-0f91-005056a772fd?countryIsoCode=gb

^3^ https://www.nice.org.uk/guidance/ng158/evidence/a-ddimer-testing-in-the-diagnosis-of-deep-vein-thrombosis-and-pulmonary-embolism-pdf-8710588334
