# Supplementary material for: Deep vein thrombosis and pulmonary embolism: a prospective, observational study to evaluate diagnostic performance of the Tina-quant D-Dimer Gen.2 assay
Source: Front Cardiovasc Med. 2023 Dec 18;10:1142465. doi: 10.3389/fcvm.2023.1142465 (PMC10759223; doi:10.3389/fcvm.2023.1142465)
Supplement: Supplementary file 1 [file Datasheet1.docx]

**Supplementary file**

**Supplementary TABLE S1 Plasma samples collected for the clinical performance evaluation of the Tina-quant D-Dimer Gen. 2 assay according to study sites and patient disposition.**

| **Cohort** | **Institution and collection site** | **Patient disposition, *N* (% total cohort)** | | | |  |
| --- | --- | --- | --- | --- | --- | --- |
|  |  | **Recruited** | **Excluded^a^** | **Lost to follow-up** | **Evaluable^b^** | |
| **DVT** | BioPartners, Inc. (Ukraine) | 1,867 (94.2) | 180 (9.1) | 14 (0.7) | 1,673 (84.4) | |
|  | Central Manchester NHS Foundation Trust (United Kingdom) | 76 (3.8) | 34 (1.7) | 3 (0.2) | 39 (2.0) | |
|  | Salford Royal NHS Foundation Trust (United Kingdom) | 37 (1.9) | 9 (0.5) | 0 (0.0) | 28 (1.4) | |
|  | Universitätsmedizin Göttingen (Germany) | 2 (0.1) | 1 (0.1) | 0 (0.0) | 1 (0.1) | |
|  | **Total** | **1,982 (100.0)** | **224 (11.4)** | **17 (0.9)** | **1,741 (87.8)** | |
| **PE** | BioPartners, Inc. (Ukraine) | 801 (87.5) | 110 (12.02) | 9 (1.0) | 682 (74.5) | |
|  | Central Manchester NHS Foundation Trust (United Kingdom) | 51 (5.6) | 12 (1.31) | 0 (0.0) | 39 (4.3) | |
|  | Universitätsklinikum Jena (Germany) | 43 (4.7) | 3 (0.3) | 0 (0.0) | 40 (4.4) | |
|  | Universitätsmedizin Göttingen (Germany) | 18 (2.0) | 5 (0.6) | 0 (0.0) | 13 (1.4) | |
|  | Salford Royal NHS Foundation Trust (United Kingdom) | 2 (0.2) | 1 (0.1) | 0 (0.0) | 1 (0.1) | |
|  | **Total** | **915 (100.0)** | **131 (14.3)** | **9 (1.0)** | **775 (84.7)** | |

^a^Patients met exclusion criteria. ^b^Patients met all inclusion and no exclusion criteria, completed the protocol and met all other evaluability criteria (e.g., all preanalytical sample handling steps were carried out appropriately, and samples and reagents were stored and shipped at the specified conditions).

DVT, deep vein thrombosis; NHS, National Health Service; PE, pulmonary embolism.

**Supplementary TABLE S2 Definitions for three-level Wells scoring: a clinical decision rule for DVT and/or PE assessment used by all study sites.**

| **Condition** | **Rules for scoring** | **Clinical characteristic (score)** |
| --- | --- | --- |
| **DVT** | - Points were given for each criterion (shown in brackets) - Scoring categories:   - >2 = high   - 1–2 = intermediate   - <1 = low | - Active cancer or cancer treated within 6 months (+1) - Paralysis, paresis, or recent casting of lower extremity (+1) - Recently bedridden (>3 days) or major surgery within the past 12 weeks (+1) - Localized tenderness in deep vein system (+1) - Swelling of entire leg (+1) - Calf swelling 3 cm greater than other leg (measured 10 cm below the tibial tuberosity) (+1) - Pitting edema greater in the symptomatic leg (+1) - Collateral non-varicose superficial veins (+1) - Prior documented history of DVT (+1) - Alternative diagnosis more or equally likely than DVT (–2) |
| **PE** | - Points were given for each criterion (shown in brackets) - Scoring categories:   - >6 = high   - 2–6 = intermediate   - <2 = low | - Clinical signs or symptoms for DVT (+3) - No alternative diagnosis better explains the illness (+3) - Tachycardia with pulse >100 bpm (+1.5) - Immobilization (≥3 days) or surgery in the previous weeks (+1.5) - Prior history of DVT or PE (+1.5) - Presence of hemoptysis (+1) - Presence of malignancy (under treatment, treated within the last 6 months, or palliative therapy) (+1) |

bpm, beats per minute; DVT, deep vein thrombosis; PE, pulmonary embolism.

**Supplementary TABLE 3 Diagnostic accuracy of the Tina-quant D-Dimer Gen.2 assay in the overall patient cohort (DVT and PE combined), by pre-test probability classification.**

|  | **True positive, *N*** | **False positive, *N*** | **False negative, *N*** | **True negative, *N*** | **NPV, %  (95% CI)** | **PPV, % (95% CI)** | **Sensitivity, %  (95% CI)** | **Specificity, %  (95% CI)** | **LR+ (95% CI)** | **LR– (95% CI)** |
| --- | --- | --- | --- | --- | --- | --- | --- | --- | --- | --- |
| **Overall cohort (DVT + PE)** | | | | | | | | | | |
| All  (*N* = 2,516) | 137 | 547 | 1^a^ | 1,831 | 100.0  (99.7–100.0) | 20.0  (17.1–23.2) | 99.3  (96.0–100.0) | 77.0  (75.3–78.7) | 4.3  (4.0–4.7) | 0.0  (0.0–0.1) |
| Low pre-test probability  (*N* = 370) | 5 | 66 | 0 | 299 | 100.0  (98.8–100.0) | 7.0  (2.3–15.7) | 100.0  (47.8–100.0) | 81.9  (77.6–85.7) | 5.5  (4.5–6.9) | 0.0  (N/A) |
| Intermediate pre-test probability  (*N* = 2,146) | 132 | 481 | 1^a^ | 1,532 | 99.9  (99.6–100.0) | 21.5  (18.3–25.0) | 99.3  (95.9–100.0) | 76.1  (74.2–78.0) | 4.2  (3.8–4.5) | 0.0  (0.0–0.1) |

^a^Female, 82 years, Caucasian; 170 cm, 69 kg; enrolled on December 5, 2018; presented directly to the ED (not by referral). No DVT lead symptoms; PE lead symptoms: tachycardia, hypotension. Vital signs: blood pressure 90/60, heart rate 120 bpm, respiratory rate 19 breaths per minute; oxygen saturation 87%; no intranasal oxygen supplementation performed; Wells score PE 4.5 (based on tachycardia and no alternative diagnosis better explaining the illness; no malignancies under treatment, treated within last 6 months or palliative therapy). Conventional 12-lead ECG; sinus tachycardia; electrical heart axis directed right; bundle branch block normal; no left-axis deviation; S1S2S3 and S1Q3R3 types: both not reported; overall ECG: non-specific (accepted deviation from the norm, with lowest likelihood of ischemia or PE).
bpm, beats per minute; CI, confidence interval; DVT, deep vein thrombosis; ECG, electrocardiogram; ED, emergency department; LR+, positive likelihood ratio; LR–, negative likelihood ratio; N/A, not applicable; NPV, negative predictive value; PE, pulmonary embolism; PPV, positive predictive value.

**Supplementary TABLE S4 Diagnostic accuracy of the Tina-quant D-Dimer Gen.2 assay in the exploratory age-adjusted analysis^a^ by decade for patients aged >50 years (*N* = 1,538).**

| **Group** | **True positive, *N*** | **False positive, *N*** | **False negative, *N*** | **True negative, *N*** | **NPV, %  (95% CI)** | **PPV, % (95% CI)** | **Sensitivity, %  (95% CI)** | **Specificity, %  (95% CI)** | **LR+  (95% CI)** | **LR– (95% CI)** |
| --- | --- | --- | --- | --- | --- | --- | --- | --- | --- | --- |
| Age >50–59 years (*N* = 534) | | | | | | | | | | |
| DVT cohort  (*N* = 395) | 9 | 39 | 1 | 346 | 99.7 (98.4–100.0) | 18.8 (9.0–32.6) | 90.0 (55.5–99.8) | 89.9 (86.4–92.7) | 8.9 (6.2–12.8) | 0.1 (0.0–0.7) |
| PE cohort (*N* = 139) | 8 | 17 | 0 | 114 | 100.0 (96.8–100.0) | 32.0 (15.0–53.5) | 100.0 (63.1–100.0) | 87.0 (80.0–92.3) | 7.7 (5.0–12.0) | 0.0 (N/A) |
| Age 60–69 years (*N* = 588) | | | | | | | | | | |
| DVT cohort  (*N* = 400) | 20 | 76 | 0 | 304 | 100.0 (98.8–100.0) | 20.8 (13.2–30.3) | 100.0 (83.2–100.0) | 80.0 (75.6–83.9) | 5.0 (4.1–6.1) | 0.0 (N/A) |
| PE cohort (*N* = 188) | 20 | 54 | 0 | 114 | 100.0 (96.8–100.0) | 27.0 (17.4–38.6) | 100.0 (83.2–100.0) | 67.9 (60.2–74.8) | 3.1 (2.5–3.9) | 0.0 (N/A) |
| Age 70–79 years (*N* = 294) | | | | | | | | | | |
| DVT cohort  (*N* = 155) | 13 | 43 | 0 | 99 | 100.0 (96.3–100.0) | 23.2 (13.0–36.4) | 100.0 (75.3–100.0) | 69.7 (61.5–77.1) | 3.3 (2.6–4.2) | 0.0 (N/A) |
| PE cohort (*N* = 139) | 21 | 47 | 3 | 68 | 95.8 (88.1–99.1) | 30.9 (20.2–43.3) | 87.5 (67.6–97.3) | 59.1 (49.6–68.2) | 2.1 (1.6–2.8) | 0.2 (0.1–0.6) |
| Age ≥80 years (*N* = 122) | | | | | | | | | | |
| DVT cohort  (*N* = 46) | 4 | 13 | 0 | 29 | 100.0 (88.1–100.0) | 23.5 (6.8–49.9) | 100.0 (39.8–100.0) | 69.1 (52.9–82.4) | 3.2 (2.1–5.1) | 0.0 (N/A) |
| PE cohort (*N* = 76) | 11 | 27 | 2 | 36 | 94.7 (82.3–99.4) | 29.0 (15.4–45.9) | 84.6 (54.6–98.1) | 57.1 (44.1–69.5) | 2.0 (1.4–2.9) | 0.3 (0.1–1.0) |

^a^An age-adjusted cut-off was determined by multiplying patient age by 0.01 µg FEU/mL (e.g., aged 55 years × 0.01 = age-adjusted cut-off of 0.55 µg FEU/mL).
CI, confidence interval; DVT, deep vein thrombosis; FEU, fibrinogen equivalent units; LR+, positive likelihood ratio; LR–, negative likelihood ratio; N/A, not applicable; NPV, negative predictive value; PE, pulmonary embolism; PPV, positive predictive value.

**Supplementary FIGURE S1** Patient flow diagram.


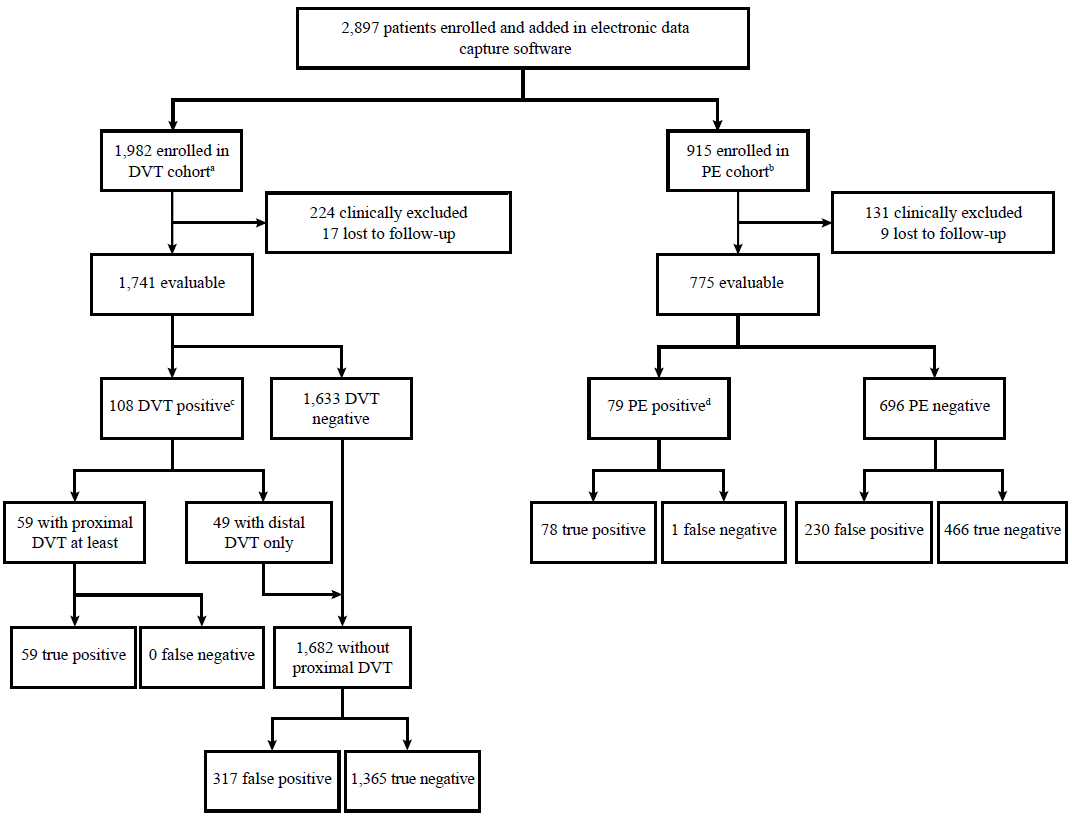


^a^Wells score indicating a low or intermediate pre-test probability for DVT (≤2) based on the respective three-level scoring, and had at least one lead symptom for DVT (or other documented reason for enrollment).

^b^Wells score indicating a low or intermediate pre-test probability for PE (≤6) based on the respective three-level scoring, and had at least one lead symptom for PE (or other documented reason for enrollment).

^c^DVT confirmed by imaging or DVT-related death at 90 days. Imaging-confirmed proximal DVT (at or above the level of the trifurcation area) was used as the primary endpoint for analysis.

^d^PE (with/without concomitant DVT) confirmed by imaging or PE-related death at 90 days. Imaging-confirmed distal PE was used as the primary endpoint for analysis.

Evaluable: participants met inclusion criteria, completed protocol, and met evaluability criteria.

DVT, deep vein thrombosis; PE, pulmonary embolism.
